# Configuring a hospital in the COVID-19 era by integrating crisis management logistics

**DOI:** 10.1017/ice.2020.365

**Published:** 2020-07-23

**Authors:** Abdulrahman Alharthy, Fahad Faqihi, Huda Mhawish, Abdullah Balhamar, Ziad A. Memish, Dimitrios Karakitsos

**Affiliations:** 1 Critical Care Department, King Saud Medical City, Riyadh, Kingdom of Saudi Arabia; 2 Research & Innovation Centre, King Saud Medical City, Riyadh, Kingdom of Saudi Arabia

*To the Editor—*The novel coronavirus SARS-CoV-2 disease (COVID-19) emerged in China and has spread throughout the world.^1^ The first case of COVID-19 in Saudi Arabia was confirmed on March 2, 2020, and presently almost 200,000 people have been infected here.^2^ The Ministry of Health (MOH) has responded to the COVID-19 outbreak by designing clusters of governmental hospitals to accommodate the increased flow of patients. Although our bed-occupancy rates never exceeded 80% until 2019, the situation has changed dramatically since March 2020, when the intensive care unit (ICU) occupancy rates reached 100% due to the pandemic. Hence, our hospital has been under pressure to upgrade our ICU services. We have used crisis management tactics in configuring our medical city (Table 1). First, we created a multidisciplinary crisis management team (CMT) to supervise the operations, and we promptly applied a surge plan based on the available scientific evidence. Our CMT policies, ICU configuration strategy, staff and resource utilization, admission protocols, and therapeutic guidelines have been reviewed continually based on new international updates, emerging therapies, and the recommendations of our national health authorities.^2–5^ By adjusting, and retrofitting existing ICUs, and acute wards. we have expanded the ICU bed capacity in a step-wise manner: phase 1, 180 beds; phase 2, 240 beds; phase 3, 300 beds). Our main challenge has been to install new structures (ie, gas access, power circuits, monitors, and HEPA purifiers) in the pop-up units. We could not maintain single-patient occupancy; thus, we isolated cohorts of COVID-19 patients in multiple-occupancy glass rooms. Nursing stations have been set up outside these rooms; new circuits have been installed for the transmission of data and alarms; and new procedure carts have been arranged for each new unit. The ICU-bed triage and staff governance have been controlled by the CMT. Our CMT members have provided coverage 24 hours per day, 7 days per week to arrange the ICU admission flow and the transfer of patients to other hospitals based on the daily MOH plan. We have followed a tiered strategy in which we allocate experienced intensivists and nurses to supervise redeployed noncritical care physicians and nurses, and we also established back-up teams. The refinement of the respiratory and ICU care included changing the ventilator circuits and filters based on patient needs, avoiding nebulizers, creating specialized intubation and prone-positioning ventilation teams, and upgrading the oxygen supply system. The latter has been a major problem for our oxygen supply management team. Hence, we have promoted awake prone positioning and more oxygen-support therapies (ie, high-flow nasal cannula, and helmet continuous positive pressure ventilation) to avoid mechanical ventilation if possible. Interventional therapies (ie, extracorporeal membrane oxygenation and therapeutic plasma exchange) have been carefully screened by expert teams to optimize resource utilization. The ICU pharmacy operations have been linked to the MOH central stock and supervised by pharmacists of the CMT to facilitate the prompt delivery of medications. Infection control measures have been strictly implemented in all hospital areas by creating specific zones and protocols for donning and doffing personal protective equipment, providing sanitizer dispensers, applying strict room-disinfection protocols, and providing safe waste handling.^6^ Moreover, we have utilized novel transportation capsule isolation technology to minimize the risk of SARS-CoV-2 acquisition during inter- and intrahospital transportation.^7^

**Table 1.** Crisis Management Tactics in Configuring a Hospital by Upgrading its Intensive Care Unit Services in the COVID-19 Era

Steps	Main challenges	Solutions
1. Launched a multidisciplinary CMT to supervise the operations	Team chemistry and communication	Enhance communication and team bonds via daily on site and virtual meetings.
2. Constant review and adjustment of CMT policies, ICU configuration strategy, staff/resource utilization, admission protocols, and therapeutic guidelines based on the international experience/recommendations, and local needs	Coordination of the flow of information	Enhance debriefing and feedback provided by all members of the team and facilitate central flow of information to avoid confusion.
3. Expansion of the ICU bed capacity in a stepwise manner based on our set-up; adjusted and retrofitted existing ICUs and acute wards.	Infrastructural issues, resource dependent process, inability to maintain single-patient occupancy	Boost new core structure development, optimize resources via the MOH command center, and launch multiple-patient glass-door rooms to cohort cases
4. ICU bed triage and staff governance	Inability of the existing system to manage the increased number of COVID-19 cases, staff shortage	Reroute admissions and transportation to other COVID-19 centers via a dedicated nursing CMT members (24/7 availability), tiered strategy for the allocation of ICU (supervising), and non-ICU staff (redeployed), and creation of backup teams to ensure constant frontline rotation
5. Refinement of the provided respiratory and ICU care	Oxygen supply issues, staff protection, shortage of resources for advanced interventional therapies	Boosting the collaboration of technical support teams, optimizing respiratory care practices, reducing mechanical ventilation strategy by boosting higher support oxygen therapies, creating intubation/awake prone positioning teams, and screening for clearance of interventional therapies (ie, ECMO) by expert teams
6. ICU pharmacy optimization pathway	Shortage and/or delivery delay of available medications	Enhance communication with central MOH stocks, and providers via dedicated CMT clinical pharmacists
7. Strict application of infection control measures	Staff awareness issues, space utilization for donning and doffing of personal protective equipment, shortage of resources, side-effects of N95 continuous utilization	Enhance staff training, application of specific protocols for infection control and proper utilization of personal protective equipment, daily supervision of all applied measures, and staff support by dedicated CMT members
8. The pivotal role of effective communication	Integration of new communication hospital systems, optimization of inter- and intrateam communication	Boosting the collaboration of technical support teams, utilizing all available free cell phone and web-based applications to ensure continuous communication
9. Training and emotional staff support	Unawareness of several COVID-19 related topics, side-effects of N95 continuous utilization, and fear of contamination, increased stress and workload	Daily dedicated training in all aspects of COVID-19 care, providing on-site care for side effects of N95 utilization, staff rotational schedule, utilizing training sessions to increase the awareness about infection control measures, and reducing healthcare worker stress

Note. CMT, crisis management team; MOH, Ministry of Health; ICU, intensive care unit; ECMO, extracorporeal membrane oxygenation.

New hospital communication systems have been installed in the pop-up ICUs. The communication between frontline staff and the CMT is continuous. Because visitors were not allowed, family meetings were organized via web-based applications to reduce patient and family stress. Training and emotional staff support have been provided on a daily basis. We have utilized daily COVID-19 training sessions to provide additional emotional reassurance (ie, dual training and emotional support strategy). Moreover, ~600 COVID-19 patients have been hospitalized in our ICU over the past 4 months. Our staff’s nosocomial infection rate was ~3% during the early stages of the pandemic, and it has decreased to 0.5% since May 2020. As the current wave of COVID-19 subsides, we are focusing on maintaining our costly infrastructure upgrades. These could ensure that a proper set-up would be available to meet future needs.^3–5^ Stores of equipment, medications, and technical gadgets remains under CMT supervision. Continuous medical education of our staff about COVID-19 by our MOH could facilitate the management of future outbreaks. Regardless of the limitations in any healthcare system, hospitals should be prepared for future pandemics.
